# Silica–boron composite nanoparticles: from architecture to mechanical performance

**DOI:** 10.1039/d6ra04393c

**Published:** 2026-07-06

**Authors:** M. Polat, M. Kaya, M. Cevik Eren, H. Paker, E. Gokmen, H. Polat

**Affiliations:** a Department of Chemical Engineering, Izmir Institute of Technology Urla-Izmir Türkiye; b Department of Chemistry, Izmir Institute of Technology Urla-Izmir Türkiye hurriyetpolat@iyte.edu.tr; c UBC Chemicals Menderes-Izmir Turkiye; d BOTEK R&D Chemistry Co. Cankaya-Ankara Türkiye

## Abstract

Controlling nanoscale architecture is critical for translating composition into functional performance in hybrid nanomaterials, yet the governing mechanisms remain poorly defined. Here, we demonstrate that precursor addition kinetics decisively govern boron incorporation and structural evolution in silica–boron nanocomposites synthesized *via* a modified Stöber process. Rapid sodium borate addition induces local supersaturation, leading to borate crystallization and phase separation, whereas controlled dosing enables homogeneous incorporation within the silica network, yielding true hybrid structures. These kinetically distinct pathways produce two fundamentally different architectures: (i) layered nanoparticles with boron-rich domains and (ii) matrix-type particles with a more homogeneous boron distribution. Comprehensive structural characterization (SEM, TEM/STEM, FTIR, XRD, EDX, and zeta potential) reveals a direct correlation between formation pathway and nanoscale organization. Crucially, these architectural differences translate into markedly different macroscopic behaviors. Layered nanostructures provide enhanced resistance to surface deformation in coating systems, while both architectures exhibit outstanding extreme-pressure performance, achieving welding loads comparable to commercial cutting oils at reduced additive concentrations. This work establishes precursor kinetics as a design lever for controlling nanoscale architecture and advancing multifunctional nanofillers with superior mechanical and tribological properties.

## Abstract 2

Controlling nanoscale architecture is critical for translating composition into functional performance in hybrid nanomaterials, yet the governing mechanisms remain poorly defined.

## Graphical abstract

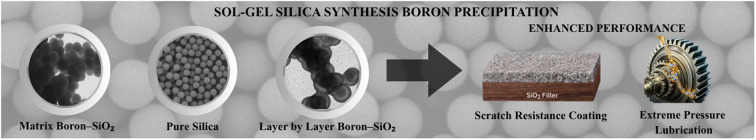

## 1 Introduction

Silica (SiO_2_) nanoparticles have been used for many years as fillers in composite materials because they are chemically stable, compatible with sol–gel processing, and suitable for surface modification.^1,2^ Among the available preparation routes, the Stöber method remains one of the most widely used approaches for producing silica particles with controlled size and morphology.^3,8^ This has made silica nanoparticles especially attractive for coating and paint formulations, where they are often used to improve surface hardness, reduce scratch damage, and strengthen the mechanical integrity of the coating layer.^9–11^ These effects are generally associated with the ability of nanosilica to limit polymer chain mobility, improve stress transfer within the matrix, and reduce localized surface deformation under mechanical loading.^12,13^ Even so, pure SiO_2_ does not always provide sufficient performance under more demanding operating conditions.^4,5,7^ This becomes more obvious in systems exposed to repeated friction, concentrated contact stresses, or severe pressure.^14,15^ In such cases, silica mainly behaves as a reinforcing phase. It can support the structure mechanically, but its role remains limited when protection is required directly at the contact interface.^16,17^ In tribological environments, for example, silica does not readily contribute to the formation of chemically active protective films, and its influence on friction and wear may therefore remain restricted, particularly under prolonged or severe service conditions.^20,21^

For this reason, interest has increasingly shifted toward hybrid filler systems that can provide not only mechanical reinforcement but also additional surface functionality.^18–22^ Boron-containing compounds are particularly attractive in this respect because of their intrinsic hardness, low-friction character, and their ability to promote the formation of boron-rich protective films under extreme-pressure conditions.^23–26^ From a fundamental materials perspective, boron can be incorporated into silica-based networks through different structural arrangements depending on precursor chemistry, pH, hydrolysis–condensation rate, and local boron concentration. In borosilicate-type systems, boron may exist as trigonal BO_3_ or tetrahedral BO_4_ units and can form Si–O–B linkages within the silicate network.^27^ These structural units can modify network connectivity, local rigidity, surface chemistry, and the distribution of active boron-containing sites. However, boron incorporation is not always straightforward in sol–gel systems. If the boron precursor is introduced too rapidly or reaches local supersaturation, borate species may undergo self-condensation or precipitation, leading to boron-rich domains rather than homogeneous integration into the silica framework. Therefore, the final behavior of boron-modified silica depends not only on the nominal boron content, but also on the competition between silica condensation, borate complexation, Si–O–B bond formation, and separate borate precipitation.^28^ In lubricant systems, boron-based additives have been reported to improve load-carrying capacity, delay seizure, and reduce wear.^29,30^ However, their direct use in polymeric or lubricating media is not straightforward. Poor dispersion stability, uncontrolled precipitation, and phase separation often reduce formulation consistency and make the final performance less reproducible.^25,26,31^

One possible way to overcome these limitations is to incorporate boron into a silica framework rather than using it as a separate additive phase.^20,21,32^ In this form, silica can provide a stable and processable structural host, while boron can contribute the functionality that pristine SiO_2_ lacks. Previous work on borosilicate and boron-modified silica materials has shown that boron incorporation can influence chemical structure, surface properties, thermal stability, hardness, scratch resistance, and tribological response.^33^ Boron-modified silica systems have also been reported to improve coating hardness, scratch resistance, and tribological behavior.^10,34,35^ These findings provide an important basis for using boron as a functional modifier in silica-based particles. However, most of the existing literature has focused mainly on the presence or amount of boron, rather than on how boron is introduced during synthesis and how that affects the final particle structure. Much less attention has been given to the precipitation behavior of the boron precursor, the mode of its addition, and the resulting internal architecture of the particles. This is a critical distinction because boron-containing silica may behave very differently depending on whether boron is homogeneously distributed within the silica network, concentrated in layered regions, or separated as borate-rich domains. Accordingly, the role of boron should be evaluated not only in terms of its total amount, but also in terms of its location, coordination environment, and distribution within the silica structure.^10,35^

In the present study, silica–boron composite nanoparticles were prepared through a modified Stöber sol–gel route in which the mode and rate of sodium borate addition were deliberately varied. The aim was not simply to add boron to the system, but to control how boron species interacted with the growing silica network and how they became incorporated into the forming particle. By changing the addition pathway, it was possible to reduce uncontrolled borate precipitation and to obtain distinct internal particle morphologies instead. In this way, two main hybrid structures were produced: one containing boron-rich layered regions and another showing a more homogeneous boron distribution throughout the silica phase. These particles were then evaluated in two different application areas, namely coating systems and oil-based formulations, in order to examine how synthesis-dependent structural differences are reflected in practical performance. In this sense, the study focuses on a straightforward but important question: not only whether boron is added, but how it is incorporated into the particle structure and how that incorporation mechanism ultimately changes the behavior of the final material.

## 2 Materials and method

### 2.1 Materials

Silica and boron–silica composite nanoparticles were synthesized using analytical-grade reagents. Tetraethyl orthosilicate (TEOS) served as the silica precursor owing to its reliable hydrolysis–condensation behavior under basic conditions, while ethanol (EtOH) provided a homogeneous reaction medium. Ammonium hydroxide (NH_4_OH) acted as the catalyst, regulating pH and enabling controlled particle growth. Sodium borate decahydrate (Na-borate) was employed as the boron source; its aqueous dissociation into borate species facilitated effective interaction with silanol groups during composite formation.

For coating formulations, a commercial varnish matrix was combined with cetyltrimethylammonium bromide (CTAB) as a surfactant and sodium hexametaphosphate (Calgon) as a dispersant to promote nanoparticle stability. Titanium dioxide (TiO_2_) was incorporated as a white pigment to aid visual evaluation of coating performance. All chemicals were obtained from certified suppliers and used without further purification.

### 2.2 Methods

The experimental procedures for synthesizing silica and silica–boron composite nanoparticles are detailed in the following sections.

#### 2.2.1 Silica synthesis: Stöber method

Silica nanoparticles were synthesized using the classical Stöber method, a sol–gel approach that enables controlled formation of monodisperse spherical particles through base-catalyzed hydrolysis and condensation of tetraethyl orthosilicate (TEOS).^36^ The synthesis was conducted in an ethanol–water medium with ammonium hydroxide serving as the catalytic base to regulate reaction kinetics.

The process involves two key steps. During hydrolysis, ethoxy groups of TEOS are replaced by hydroxyl groups, forming silanol species:

1 Si(OC_2_H_5_)_4_ + 4H_2_O → Si(OH)_4_ + 4C_2_H_5_OH

Subsequently, condensation reactions between silanol groups yield siloxane (Si–O–Si) bonds, generating a three-dimensional silica network:

2 Si–OH + HO–Si → Si–O–Si + H_2_O

**Fig. 1:**
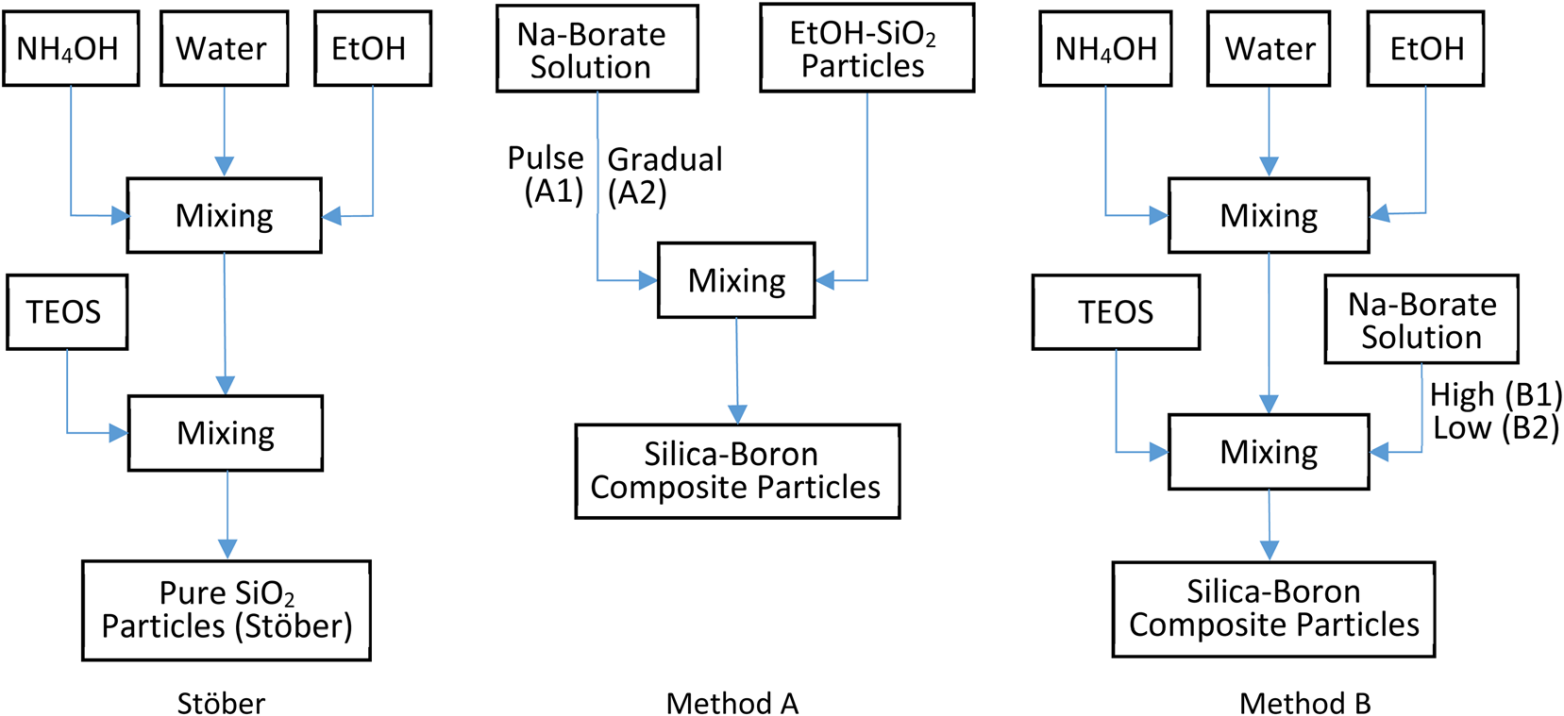
Flow chart of all methods to synthesize nanoparticles.

Once supersaturation is reached, homogeneous nucleation occurs, followed by particle growth through surface condensation of hydrolyzed species. The balance between nucleation and growth determines the final particle size, which can be tuned by adjusting TEOS concentration, ammonia content, and solvent composition. A schematic flowchart summarizing the Stöber synthesis steps is provided in Fig. 1.

#### 2.2.2 Silica–borate synthesis methods

Silica–boron composite nanoparticles were synthesized using a modified Stöber approach employing two distinct structural design strategies (Method A and Method B), each comprising two kinetic variants (A1/A2 and B1/B2). Precursor addition kinetics were deliberately varied to regulate boron incorporation and internal particle architecture (Fig. 1).

##### 2.2.2.1 Method A

Monodisperse SiO_2_ nanoparticles were first synthesized *via* the classical Stöber process and dispersed in ethanol to form a stable colloidal suspension. An aqueous sodium borate (Na–B) solution was then introduced to deposit boron onto the pre-formed silica particles. In Method A1 (pulse addition), Na–B was added rapidly, producing local supersaturation that favored borate crystallization as a separate phase, with silica particles attaching to borate domains. In Method A2 (gradual addition), Na–B was introduced continuously using a syringe pump, enabling controlled deposition and more homogeneous incorporation of boron onto silica surfaces. These variants provided precise control over boron loading while maintaining silica monodispersity.

##### 2.2.2.2 Method B

In Method B, Na–B was added directly into the Stöber reaction medium during silica particle growth, enabling periodic interaction between boron species and surface silanol groups. This promoted interactions between boron species and surface silanol groups, potentially facilitating the formation of Si–O–B linkages. In Method B1 (high Na–B concentration), alternating boron-rich and silica-rich regions developed, yielding a layered composite architecture. In Method B2 (low Na–B concentration), boron was incorporated more homogeneously throughout the silica framework, producing a homogeneous matrix-type composite.

These synthesis routes systematically modulate boron incorporation kinetics and internal architecture, and the resulting structural differences are examined in detail in the subsequent sections. The structural outcomes of these synthesis routes—ranging from phase-separated domains to layered and matrix-type hybrids—are examined in detail in the following Results and Discussion section, beginning with the characterization of pristine silica and borate reference materials.

#### 2.2.3 Applications with silica and silica–boron composite nanoparticles

For all coating applications containing silica and silica–boron composite nanoparticles, the experimental procedures were carried out under identical conditions to ensure reliable comparison between the samples. Performance measurements, including scratch resistance and tribological tests, were repeated three times for each sample, and the reported values were presented as the average of these replicate measurements. This approach was followed to improve the reproducibility and reliability of the experimental results.

##### 2.2.3.1 Silica–boron composite nanoparticles in oil-based systems

To evaluate the applicability of silica–boron composite nanoparticles in oil-based systems, nanoparticle-containing lubricant formulations were prepared and characterized prior to tribological testing. The experimental procedure focused on achieving stable dispersion of the composite nanoparticles within a mineral oil matrix and assessing their performance under extreme pressure conditions.

Mineral base oil (Base Oil-1 obtained from OPET) was selected as the carrier fluid. Boron–SiO_2_ composite nanoparticles were added to the oil at a % 5 weight fraction. CTAB was used as a cationic surfactant to improve the dispersion stability of the formulation.^35^ Afterward, the mixtures were sonicated for a fixed time to obtain a more homogeneous distribution of nanoparticles within the oil phase. The resulting dispersions were then observed over a certain period for any signs of sedimentation or phase separation.

The extreme pressure (EP) performance of the oil formulations was evaluated using the Four–Ball test method. To systematically examine the effect of boron–SiO_2_ composite nanoparticles on EP behavior, the formulations were prepared and tested at three different additive loadings as 0.15 (Design 1), 0.20 (Design 2) and 0.30 (Design 3) weight%, while all other formulation parameters were kept constant. Neat base oil and commercial cutting oil were included as reference samples. The test was conducted using a standard four-ball configuration with three stationary steel balls and one rotating steel ball fully immersed in the test oil. During the experiment, the applied load increased stepwise at a constant rotational speed until welding occurred at the contact interface, and the welding load was recorded as the primary indicator of EP performance.

After the Four–Ball EP tests, the surfaces of selected stationary balls were examined using a computer-connected optical microscope at OPET Fuchs. Representative images were recorded to illustrate different wear conditions, including an unworn control surface, and a severely worn surface. The wear scar diameter observed on the worn samples was approximately 0.4 mm, depending on the severity of surface damage.

##### 2.2.3.2 Silica–boron composite nanoparticles in paint coating systems

The silica and silica–boron composite nanoparticles were incorporated into paint formulations a commercial varnish matrix was selected as the coating medium to provide a representative organic binder system commonly used in industrial paint applications. The use of the same matrix for all formulations ensured that performance differences could be directly attributed to the type and structure of the incorporated nanoparticles. An example paint mixture formulation given below was the combination of the formulations taken from the Paint Companies from Izmir-Turkey (Kanat Paints and Coatings, Kansai Altan): A binder (35%), solvent (50%), pigment (1%), filler material (10%) and additives (4%).

Within the additives fraction, appropriate dispersion aids were incorporated to improve the wettability of inorganic particles and to suppress agglomeration, thereby ensuring a stable and uniform distribution throughout the paint system. The nanoparticle phase, including pure SiO_2_ and silica–boron composite structures, was introduced as part of the filler material fraction, contributing to the overall solid content of the formulation.

The pigment component (1 wt%) was included to provide opacity and to enable clear visualization of surface damage during scratch resistance testing. The prepared paint mixtures were subjected to short-duration ultrasonication to enhance dispersion quality and minimize the formation of aggregates that could negatively influence coating performance.

Subsequently, the formulations were applied onto solid substrates using a standardized applicator technique to ensure uniform coating thickness. The coated samples were then allowed to dry under ambient conditions prior to further mechanical and surface characterization.

Scratch resistance was first assessed using the pencil hardness method as a rapid screening tool to rank coating hardness. The test was carried out according to ASTM D3363, using pencils spanning 9B (soft) to 9H (hard). Each pencil was fixed in a holder at 45° to the coated substrate and moved 6.5 mm across the surface to generate a controlled scratch. The standard practice was followed by starting from the hardest pencil (9H) and proceeding to softer grades if visible damage occurred, defining the coating hardness as the hardest pencil that does not produce scratching or film damage.^37^

To mimic more practical, “daily-use” mechanical damage and to enable direct visual comparison between formulations, an applicator-based scratch test was also performed. A constant, repeatable force was applied with an applicator to create five horizontal and five vertical crossing lines on each coated sample. The goal was to keep the scratch lines comparable in strength so that differences in mechanical resistance could be judged consistently, and the scratch behavior of coatings containing SiO_2_*versus* Boron–SiO_2_ composites could be compared side-by-side.

Scratch-induced surface damage was supported by roughness measurements using a profilometer to quantify changes in surface topography. In this approach, a diamond-tipped stylus was moved across the coating surface to collect texture/roughness data, reported as Ra (roughness average). Ra was treated as an indicator of surface smoothness based on the average absolute deviation of the surface profile from a baseline, where higher Ra values correspond to rougher surfaces.

## 3 Results & discussion

### 3.1 Synthesis and characterization of pristine silica nanoparticles and boron structures

Silica nanoparticles synthesized *via* the Stöber method and sodium borate crystals precipitated in ethanol solution were characterized by SEM, FTIR, and XRD to establish a reference framework for evaluating boron–silica composites. This baseline characterization defined the intrinsic morphology and structure of pristine phases, ensuring that subsequent changes observed in composite systems could be rigorously assessed.

**Fig. 2:**
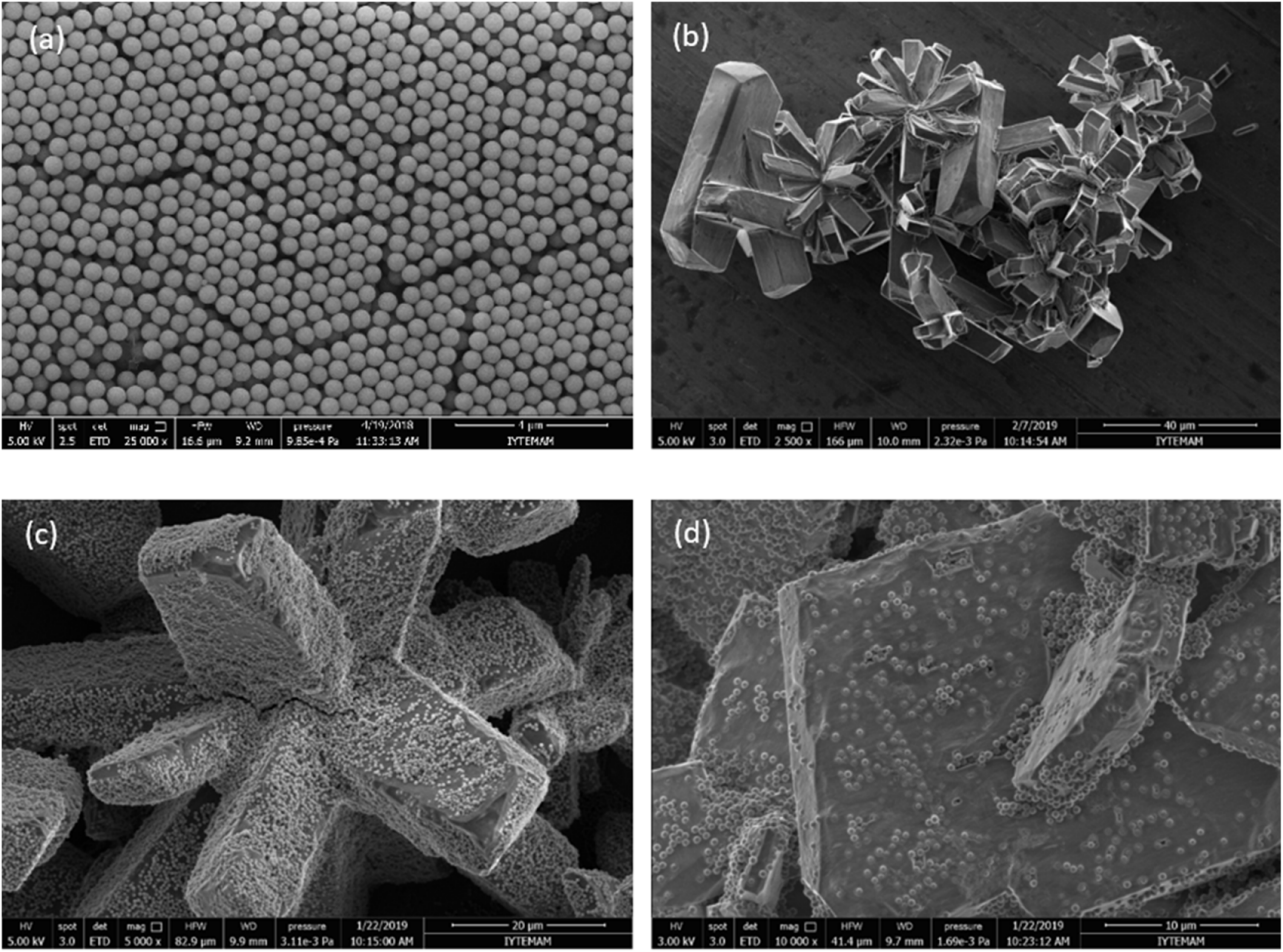
Synthesis methods for forming composite silica–boron nanoparticles. (a) pure Stöber silica; (b) pure Na–borate precipitate; (c) silica–boron nanoparticles produced by Method A1 (d) silica–boron nanoparticles produced by Method A2.

SEM images of pristine silica nanoparticles (Fig. 2a) revealed uniform spherical morphology with smooth, well-defined surfaces. The particles were <1 µm in diameter, monodisperse, and exhibited a narrow size distribution, indicating effective control of nucleation and growth typical of a well-regulated Stöber synthesis.^38^ Variations in TEOS concentration influenced average particle size but did not compromise morphological integrity.

**Fig. 3:**
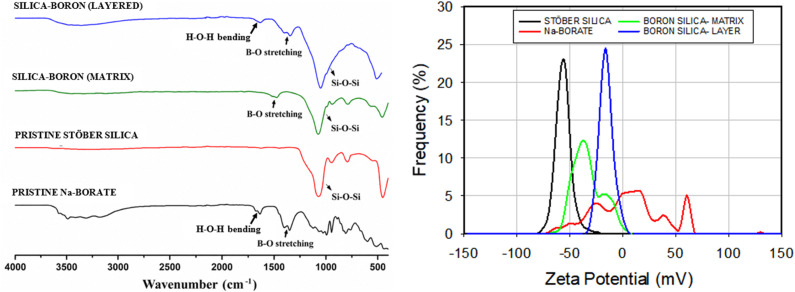
FTIR spectrum and zeta potential of Stöber silica, Na–borate and silica–boron composite (Method B1 and B2) particles.

**Fig. 4:**
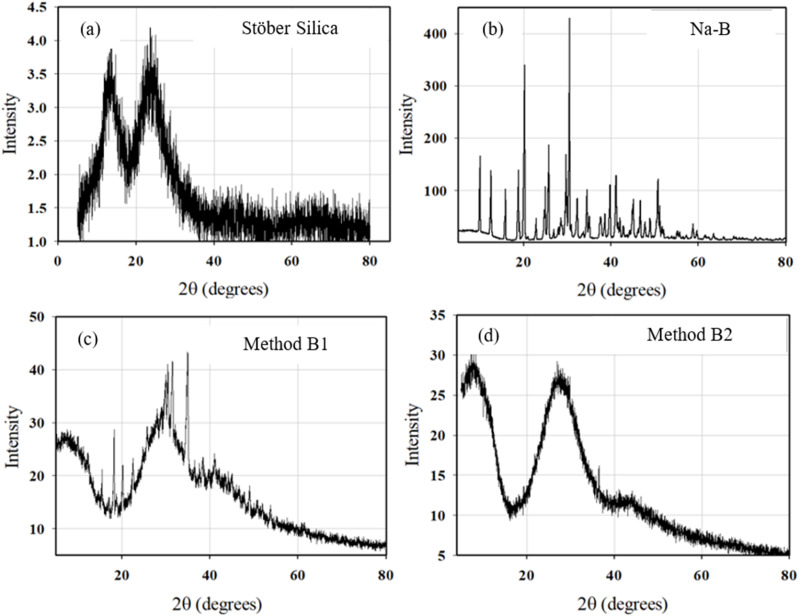
XRD scans of (a) pristine Stöber silica particles, (b) sodium borate crystals, (c) silica–boron composite particled produced by B1, (d) silica–boron composite particles produced by B2.

FTIR spectra (Fig. 3) displayed characteristic Si–O–Si stretching vibrations at ∼450 cm^−1^ and 1000–1100 cm^−1^, confirming silanol condensation into a pure silica network. No secondary phase bands were detected. Zeta potential measurements showed a narrow distribution centered at −55 mV, consistent with strongly negative surface charge. XRD scans (Fig. 4) exhibited broad twin peaks characteristic of amorphous silica.

SEM images of sodium borate precipitates (Fig. 2b) revealed large crystalline domains, tens of micrometers in size, often forming clusters. FTIR spectra (Fig. 3) showed prominent bands for O–H stretching (∼3400 cm^−1^), H–O–H bending (∼1650 cm^−1^), and strong B–O stretching (∼1400 cm^−1^), confirming trigonal BO_3_ units. Additional broad features below 1000 cm^−1^ corresponded to B–O–B and B–O–Si linkages. Zeta potentials spanned −75 mV to +75 mV with a mean near 0 mV, reflecting heterogeneous surface charging. XRD scans (Fig. 4) displayed sharp peaks characteristic of crystalline borate phases.

### 3.2 Synthesis and characterization of silica–boron composite nanoparticles

Silica–boron composite nanoparticles were synthesized using two distinct approaches (Methods A and B; Fig. 1). In Method A, sodium borate (Na–borate) was added to a dispersion of pre-formed silica nanoparticles, whereas Method B involved the gradual introduction of Na–borate into the Stöber reaction medium during particle growth, enabling direct interaction between boron species and hydrolyzed TEOS intermediates. Each approach was further investigated using two addition modes (A1/A2 and B1/B2) to evaluate the influence of addition kinetics on composite formation.

In Method A1, Na–borate was introduced rapidly as a pulse into the ethanol–water dispersion containing silica nanoparticles. The resulting local supersaturation promoted the homogeneous nucleation and crystallization of sodium borate as a separate phase rather than its controlled deposition onto silica surfaces (Fig. 2c). Consequently, silica nanoparticles adhered physically to the surfaces of borate crystals, indicating limited heterogeneous nucleation due to the insufficient availability of reactive silanol sites.

In contrast, Method A2 employed gradual Na–borate addition using a micro-syringe pump, which suppressed instantaneous supersaturation and facilitated progressive interactions between borate species and surface silanol groups. This approach produced composite structures in which silica nanoparticles became embedded within borate crystals (Fig. 2d). Nevertheless, borate still crystallized as a distinct phase rather than forming continuous coatings around individual silica particles. Because neither A1 nor A2 yielded the targeted core–shell architecture, further characterization by FTIR, zeta potential, and XRD was not pursued for these samples.

Despite these limitations, the results obtained using Method A clearly demonstrate that the mode of Na–borate addition strongly influences local supersaturation, the nucleation kinetics of borate species, and their interfacial interactions with pre-formed silica particles. These factors, in turn, govern phase evolution and the resulting particle morphology in boron-modified silica systems. The observed behavior is consistent with previous reports on silica–borate composites^6,26^ and highlights the importance of kinetic control in tailoring boron distribution and nanoparticle architecture.

**Fig. 5:**
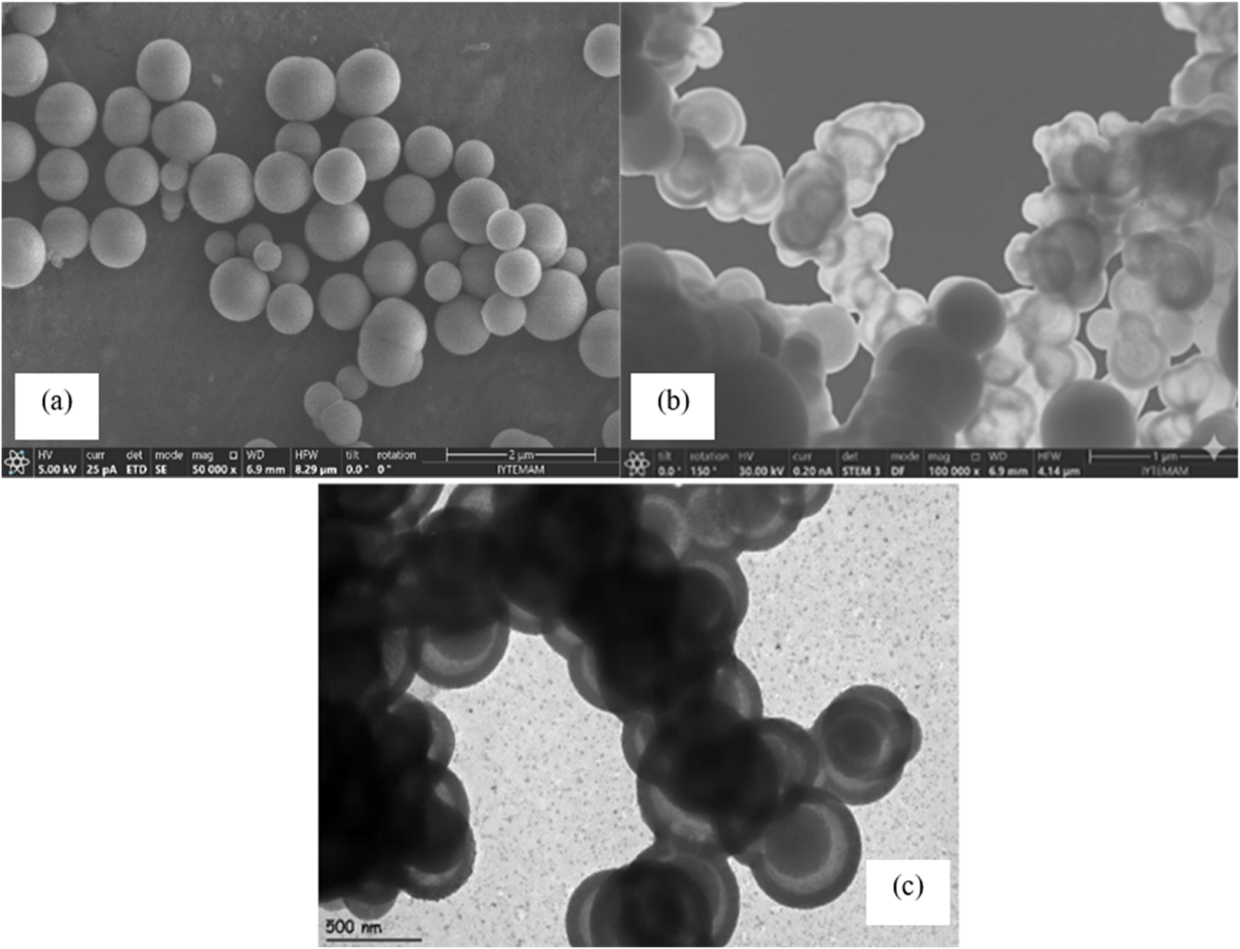
(a) SEM, (b) STEM and (c) TEM images of the silica boron particles obtained by Method B1.

**Fig. 6:**
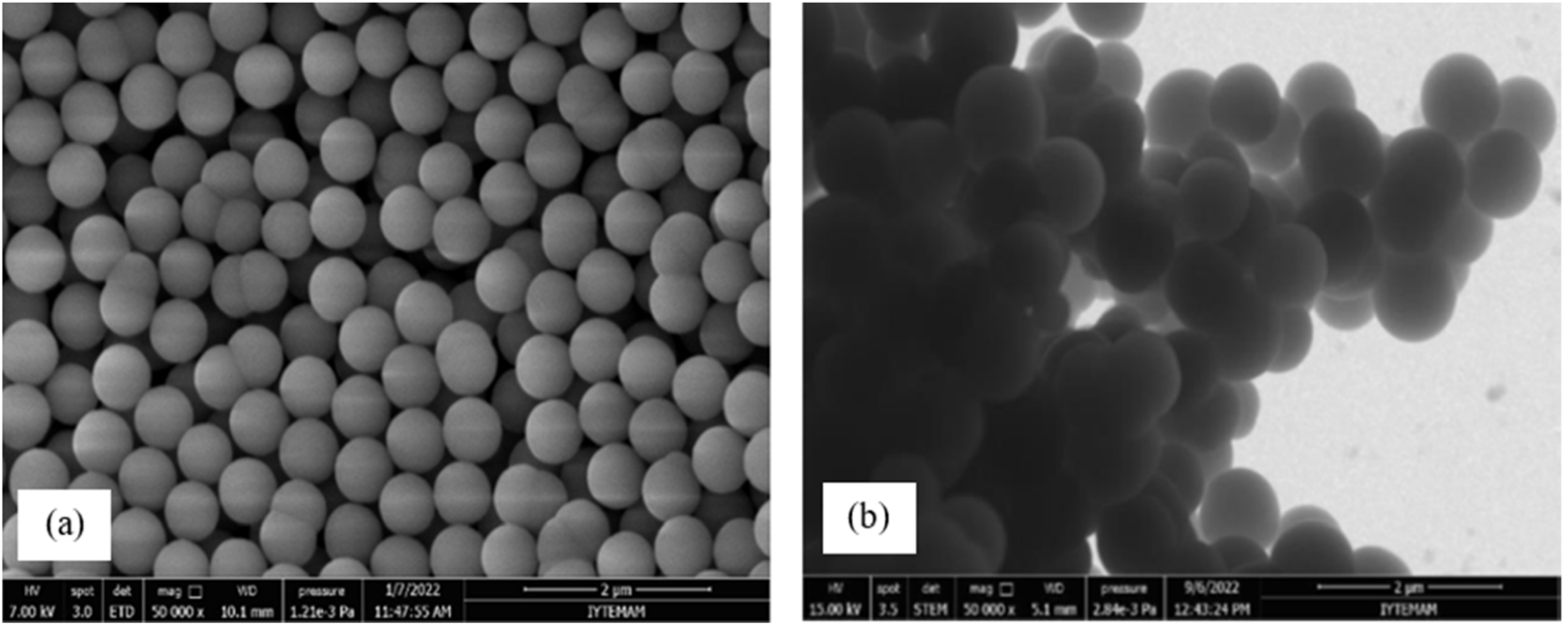
(a) SEM and (b) STEM images of silica–boron nanoparticles *via* method B2.

In Method B1, Na–borate was introduced at a relatively high concentration. SEM, STEM, and TEM analyses (Fig. 5) revealed particles with irregular morphologies and rougher surfaces compared with pristine silica nanoparticles. TEM images further demonstrated a layered composite architecture consisting of alternating boron-rich and silica-rich domains. In contrast, Method B2 employed sub-stoichiometric Na–borate concentrations, yielding particles that closely resembled pristine silica. These particles exhibited homogeneous, amorphous structures with no evidence of layering or distinct phase separation between silica and boron species, even under high-resolution TEM examination (Fig. 6).

The structural differences observed by electron microscopy were further corroborated by FTIR spectroscopy (Fig. 3). All composites exhibited the characteristic Si–O–Si stretching vibrations of the silica framework (∼1100–1000 cm^−1^) together with B–O stretching bands in the ∼1450–1350 cm^−1^ region, supporting the successful incorporation of boron species into the silica matrix. The B–O band, absent in pristine Stöber silica, is commonly attributed to the asymmetric stretching vibrations of trigonal BO_3_ units. In borosilicate systems, however, this region may also include contributions from Si–O–B linkages and borate species interacting with surface silanol groups.

A distinct band at ∼1630–1650 cm^−1^, assigned to the bending vibration of molecularly adsorbed water, *δ*(H–O–H), provided additional insight into the distribution of boron species within the composites. The layered B1 structure exhibited a pronounced *δ*(H–O–H) band together with stronger B–O absorption, consistent with a boron-rich, hydrated outer region containing residual B–OH groups and entrapped water molecules. In contrast, the matrix-type B2 composite displayed weaker and broader B–O features accompanied by minimal water-related absorption, consistent with a more homogeneous distribution of boron species throughout the silica network. Collectively, these spectral differences confirm the formation of two distinct composite architectures—a layered silica–boron structure and a matrix-type composite—rather than a simple physical mixture of silica and sodium borate.

The distinct interfacial structures of the two composites were also reflected in their surface charge characteristics. Zeta potential measurements (Fig. 3) narrowed to approximately −15 mV for the layered B1 composite, indicating reduced surface charge due to boron enrichment at the particle interface. In contrast, the B2 matrix composite exhibited zeta potentials ranging from −55 to +5 mV, with an average value of approximately −35 mV, placing its electrophoretic behavior between that of pristine silica and sodium borate crystals.

XRD analysis (Fig. 4) provided further evidence for the different modes of boron incorporation. The B1 layered composite exhibited sharp borate diffraction peaks superimposed on the broad amorphous halo of silica, confirming the presence of boron-rich crystalline domains. By comparison, the B2 matrix composite displayed only broad amorphous silica features with no detectable crystalline borate phases, supporting the predominantly amorphous nature of this structure and its proposed matrix-type architecture.

**Fig. 7:**
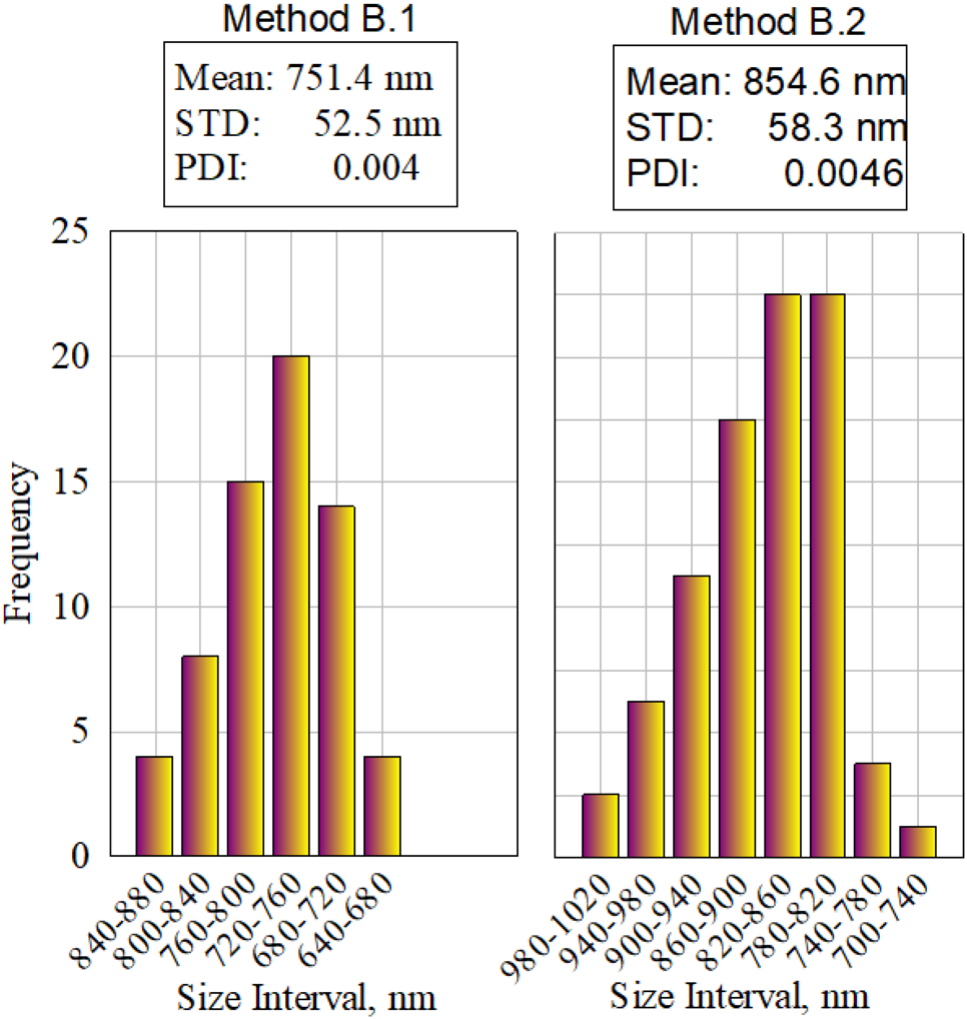
Size distribution histograms of silica–boron composite nanoparticles synthesized by Methods B1 and Method B2.

Particle size analysis (Fig. 7) revealed narrow size distributions for both composite architectures. The layered B1 composite exhibited particle diameters ranging from 640 to 840 nm, with a mean size of 751 nm and an exceptionally low polydispersity index (PDI = 0.004), indicating highly controlled particle growth despite its internal heterogeneity. The B2 matrix composite displayed a slightly larger size distribution (700–1020 nm; mean diameter = 855 nm; PDI = 0.046), consistent with continuous particle growth facilitated by the homogeneous incorporation of boron species throughout the silica matrix.

**Fig. 8:**
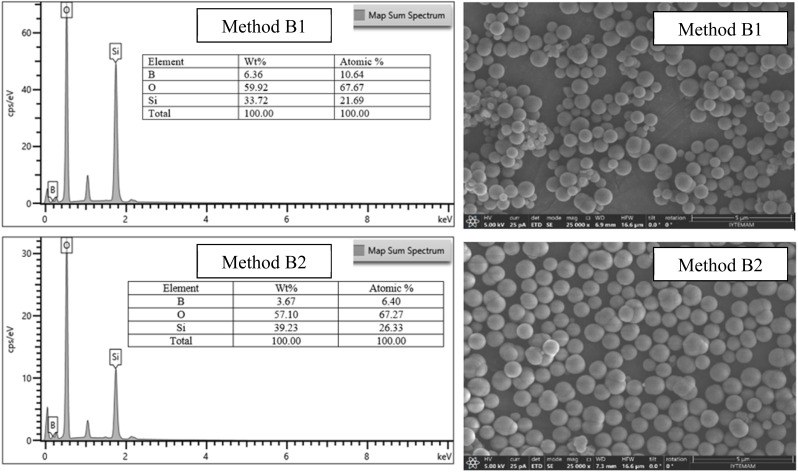
EDX analysis of the elements present in Method B1 and Method B2 and along with the STEM images of the particle populations subjected to the EDX scans.

Although high-resolution TEM did not reveal distinct silica–boron interfaces or compositional fringes within the B2 particles, the combined FTIR, zeta potential, and XRD results strongly suggested the successful incorporation and homogeneous distribution of boron throughout the silica framework. To further validate this interpretation, SEM-EDX analyses were performed on both the B2 matrix composite and the layered B1 particles (Fig. 8).

STEM-EDX measurements confirmed distinct boron incorporation pathways for the two synthesis strategies. Both composites exhibited strong boron signals; however, their Si ratios differed markedly. The layered B1 structure displayed an approximate Si ratio of 2 : 1 (Si = 33.72 wt%, *B* = 6.36 wt%), indicative of boron surface enrichment and the presence of segregated borate domains. In contrast, the B2 matrix composite exhibited a higher Si ratio of approximately 4 : 1 (Si = 39.23 wt%, *B* = 3.67 wt%), consistent with the homogeneous dispersion of boron species within the silica network.

These observations are in agreement with previous studies on sol–gel-derived borosilicates, which have reported homogeneous boron incorporation below approximately 10 mol% B_2_O_3_, corresponding to Si ratios in the range of 8–15 : 1.^39,40^ Although the boron contents observed here are higher, the trend remains consistent: elevated Na–borate concentrations favor boron-rich surface domains and phase segregation, whereas sub-stoichiometric borate addition promotes progressive Si–O–B bond formation and more uniform boron incorporation during particle growth.

Collectively, these findings demonstrate that the synthesis strategy critically governs the internal architecture of silica–boron composite nanoparticles. Method B1, employing high Na–borate concentrations, promotes layer-by-layer growth, localized boron enrichment, and structural heterogeneity. In contrast, Method B2, based on sub-stoichiometric borate addition, yields a predominantly amorphous matrix structure with homogeneous boron distribution. This clear mechanistic distinction between layered and matrix-type architectures provides a robust framework for interpreting the filling and coating performance of the composites discussed in the following sections.

### 3.3 Scratch resistance effect of nanoparticles in paint

The practical impact of incorporating silica and silica–boron composite nanoparticles into paint formulations was evaluated through scratch resistance testing supported by surface characterization. With binder composition and application conditions held constant across all samples, differences in surface durability can be attributed primarily to the nanoparticle reinforcement—specifically the internal architecture of the boron–silica composites (layer-by-layer *vs.* matrix-type).

**Fig. 9:**
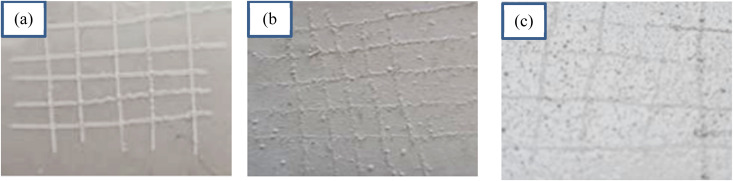
Surface scratch test results of coatings formed by composite nanoparticles (a) pristine silica, (b) silica–boron composite (B1), (c) silica–boron composite (B2).

Scratch resistance was qualitatively assessed using applicator-based scratching and pencil hardness testing. Coatings containing pure SiO_2_ exhibited pronounced scratch marks, indicating limited mechanical protection under the test conditions. In contrast, coatings reinforced with silica–boron composites showed markedly reduced surface damage (Fig. 9). The strongest improvement was observed with particles synthesized by Method B1, which produced shallower, less pronounced scratches. Particles from Method B2 also enhanced scratch resistance relative to SiO_2_, though the effect was less pronounced than in the layered B1 architecture.

Profilometric measurements (Fig. 9) corroborated the scratch testing results. The SiO_2_-filled coating exhibited a post-scratch surface roughness (*R*_a_) of 2.4 µm. In contrast, coatings containing silica–boron composites showed significantly lower roughness values, consistent with improved resistance to pitting and microscratching. Method B2 yielded an *R*_a_ of 1.8 µm, while Method B1 consistently produced the lowest roughness (1.1 µm). These results suggest that the layered B1 architecture provides superior protection against permanent surface damage, likely due to boron-rich outer regions imparting enhanced hardness and resistance during mechanical contact.

Overall, scratch and surface characterization results demonstrate that boron–SiO_2_ composites offer a clear advantage over conventional silica nanoparticles as functional fillers in scratch-resistant coatings. Importantly, composite architecture is decisive: the layer-by-layer design (Method B1) provides the most robust surface protection, whereas the matrix-type design (Method B2) delivers moderate yet consistent improvement. These observations establish a direct link between nanoscale architecture and macroscopic coating performance (Fig. 9), providing a strong rationale for architecture-driven synthesis strategies in advanced protective coatings.

### 3.4 Tribological performance of composite nanoparticles in oil-based systems

**Fig. 10:**
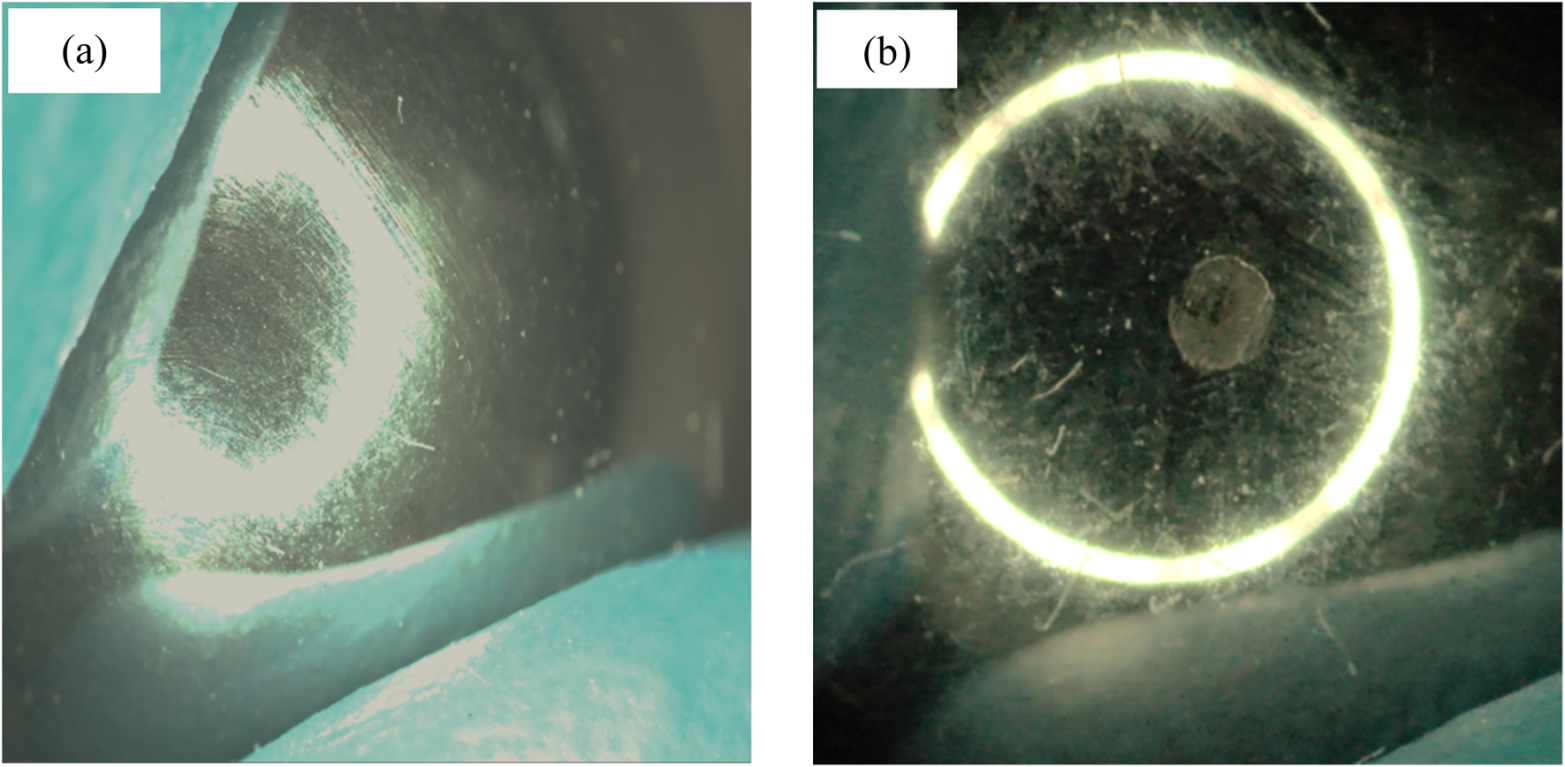
Four-ball testing results after testing with neat oil (a) the control ball surface (no abrasion), (b) abraded ball (0.4 mm).

The tribological performance of silica–boron composite nanoparticles synthesized by Method B2 was evaluated in oil-based systems using Four–Ball EP testing. The neat base oil exhibited a welding load of 1000 N, which increased progressively with nanoparticle loading, reaching 8000 N at the highest concentration—comparable to commercial cutting oil. Representative ball surface images before and after testing in the absence of nanoparticles (Fig. 10) illustrate baseline abrasion.

Direct visualization of tribofilm formation in the presence of silica–boron nanoparticles using SEM or optical microscopy proved difficult, likely due to the thin and discontinuous nature of the film resolved. However, the pronounced increase in welding load with rising nanoparticle concentration is consistent with the involvement of tribochemical processes. The enhanced performance is therefore interpreted as consistent with tribofilm formation, in agreement with prior reports on boron-containing additives. Definitive confirmation of tribofilm composition would require advanced techniques (*e.g.*, XPS, TEM, Raman), which were beyond the scope of this study.

As expected, the neat base oil exhibited a low welding load (1000 N), reflecting limited load-carrying capacity in the absence of EP-active additives. By contrast, the commercial cutting oil achieved 8000 N, consistent with optimized industrial EP formulations. Incorporation of silica–boron composite nanoparticles into the base oil produced a progressive improvement in EP performance with increasing additive concentration.

At the lowest additive concentration (Design 1), only a marginal increase in welding load (1260 N) was observed, indicating insufficient nanoparticle content to form a continuous protective interface. At intermediate loading (Design 2), the welding load rose substantially to 6200 N, reflecting improved seizure resistance and more effective load transfer at the contact zone. At the highest concentration (Design 3), the welding load reached 8000 N—matching commercial cutting oil performance despite the markedly lower additive content in the composite-based formulation.

These results suggest the existence of a critical additive loading above which composite nanoparticles actively contribute to tribofilm formation and surface protection. The tribological performance can be attributed to the synergistic action of the silica matrix and boron species. The silica phase enhances mechanical stability, smooths the contact surface, and distributes load more evenly, while boron species become tribochemically active under high pressure, promoting boron-rich protective films. These films reduce direct metal-to-metal contact and delay the onset of welding.

Collectively, these findings indicate that silica–boron composite nanoparticles function as highly effective extreme-pressure additives in oil-based systems. Even at low concentrations, they deliver tribological performance comparable to commercial formulations. This highlights the promise of architecture-controlled composite nanoparticles as a practical route to high-performance lubricant additives, with the added benefit of reducing additive dosage requirements.

## 4 Conclusions

This study establishes architecture-driven synthesis as a decisive strategy for designing advanced boron–silica nanofillers. By controlling sodium borate incorporation within a modified Stöber sol–gel route, precipitation kinetics were directly linked to nanoparticle architecture and performance. Rapid, uncontrolled addition produced phase-separated morphologies, whereas controlled pathways yielded true hybrids with either layered or matrix-type structures. These architectures governed macroscopic behavior: layered designs maximized scratch resistance in coatings, while matrix-type hybrids provided uniform durability; in oil-based systems, both architectures delivered extreme-pressure protection through synergistic silica stabilization and tribochemically active boron species. These findings demonstrate that deliberate control of nanoscale architecture translates into reliable, enhanced performance across diverse applications, offering a framework for next-generation composite nanofillers.

## Author contributions

H. Polat and M. Polat conceptualized the study and supervised the research. M. Kaya, M. C. Eren, H. Paker and E. Gökmen performed experiments/data analysis. H. Polat drafted the manuscript with input from all authors. All authors reviewed and approved the final version.

## Conflicts of interest

There are no conflicts to declare.

## Funding

This research was funded by İzmir Institute of Technology Scientific Research Projects Fund (BAP) under the title “IYTE0213 Synthesis of Composite (Silica–Sodium Borate) Nanoparticles with Different Size Distributions”

## Data Availability

The data supporting this study are available from the corresponding author upon reasonable request.
